# Supplementary figures and images for: Plasmodium falciparum expresses fewer var genes at lower levels during asymptomatic dry season infections than clinical malaria cases
Source: PLoS Pathog. 2025 Jun 10;21(6):e1013210. doi: 10.1371/journal.ppat.1013210 (PMC12151486; doi:10.1371/journal.ppat.1013210)

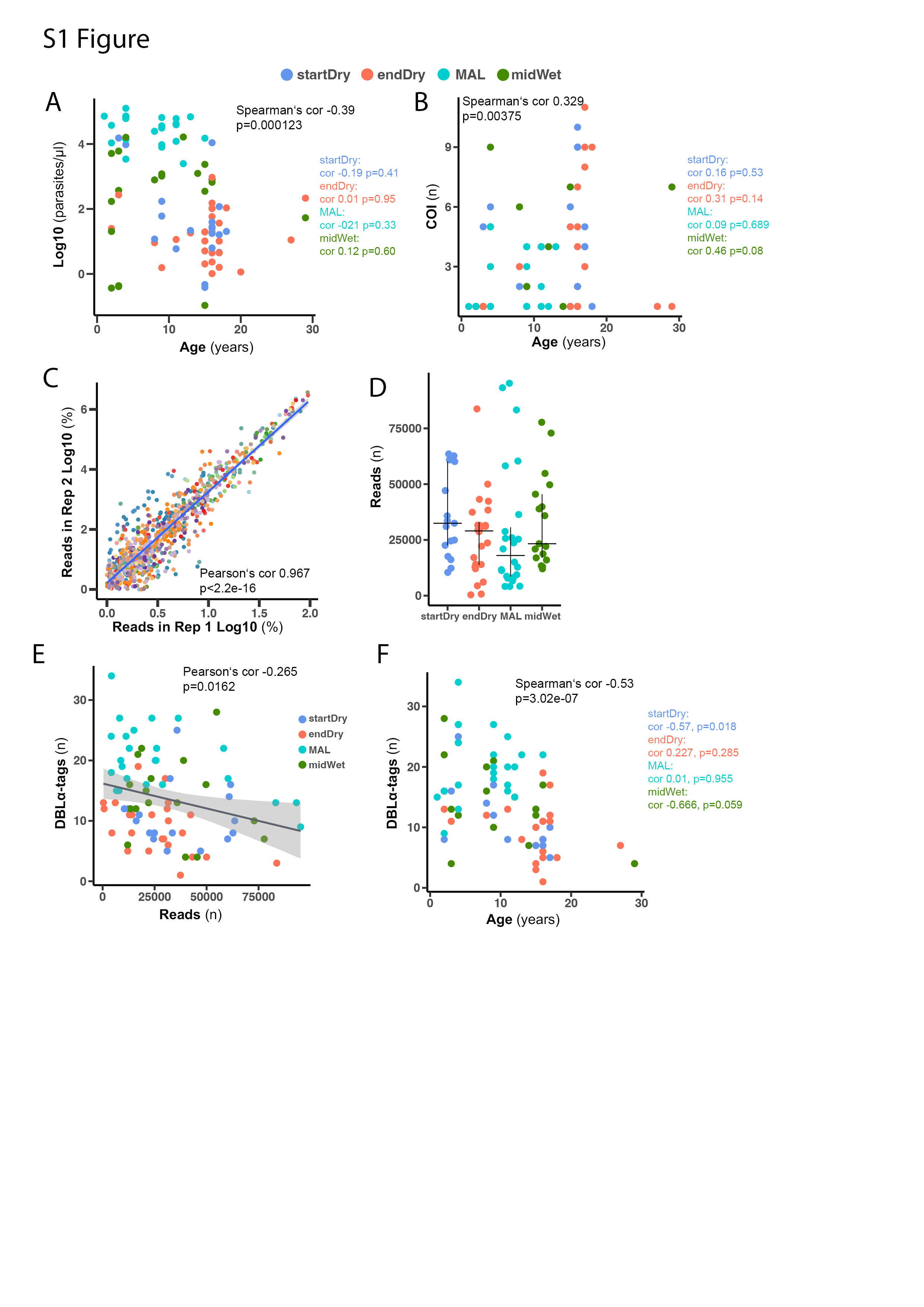

Supplement: S1 Fig — (A) Correlation of parasitaemia and participant age in October 2019 in 91 samples from beginning of the dry season (startDry n = 30), end of the dry season (endDry n = 28) and clinical malaria cases (MAL n = 24) and the wet season (midWet n = 22). (B) Correlation of complexity of infection (COI) and participant age in 79 (startDry (n = 20), endDry (n = 23) and midWet (n = 15)) asymptomatic infections and clinical malaria cases (MAL n = 21). (C) Correlation between DBLα-tag abundance between clusters in 60 samples with duplicates of > 500 reads. (D) Read count in 82 samples (startDry = 17, endDry n = 24, MAL n = 24, midWet n = 17) from the four timepoint after quality filtering. (E) Correlation between read count and number of DBLα-tags in a sample with color indicating timepoint. (F) Correlation between number of DBLα-tag clusters in the same samples and participant age with color indicating timepoint. (TIF) [file ppat.1013210.s001.tif]

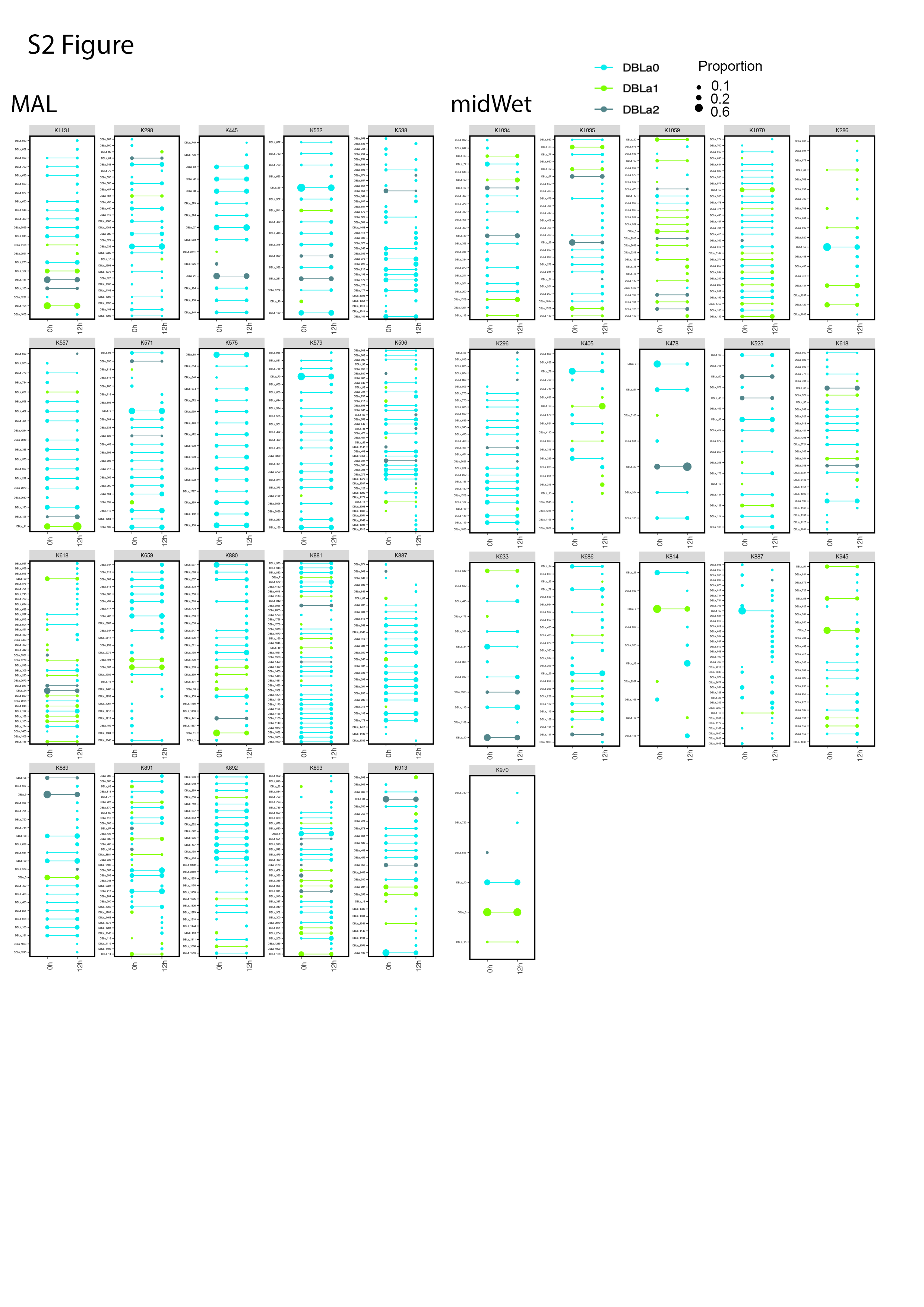

Supplement: S2 Fig — Comparison of DBLα-tags in 16 wet season asymptomatic (midWet) samples (left) and 20 MAL (right) ex vivo samples (0h) and the corresponding short-term culture timepoints (12h). Individual clusters are plotted on the y-axis with lines connecting clusters detected at the 0h and 12h timepoint. Dot size indicates proportion of reads in sample mapping to the cluster. DBLα-tags were annotated with DBLα subtype by best BLAST hit in a database of annotated var genes. (TIF) [file ppat.1013210.s002.tif]

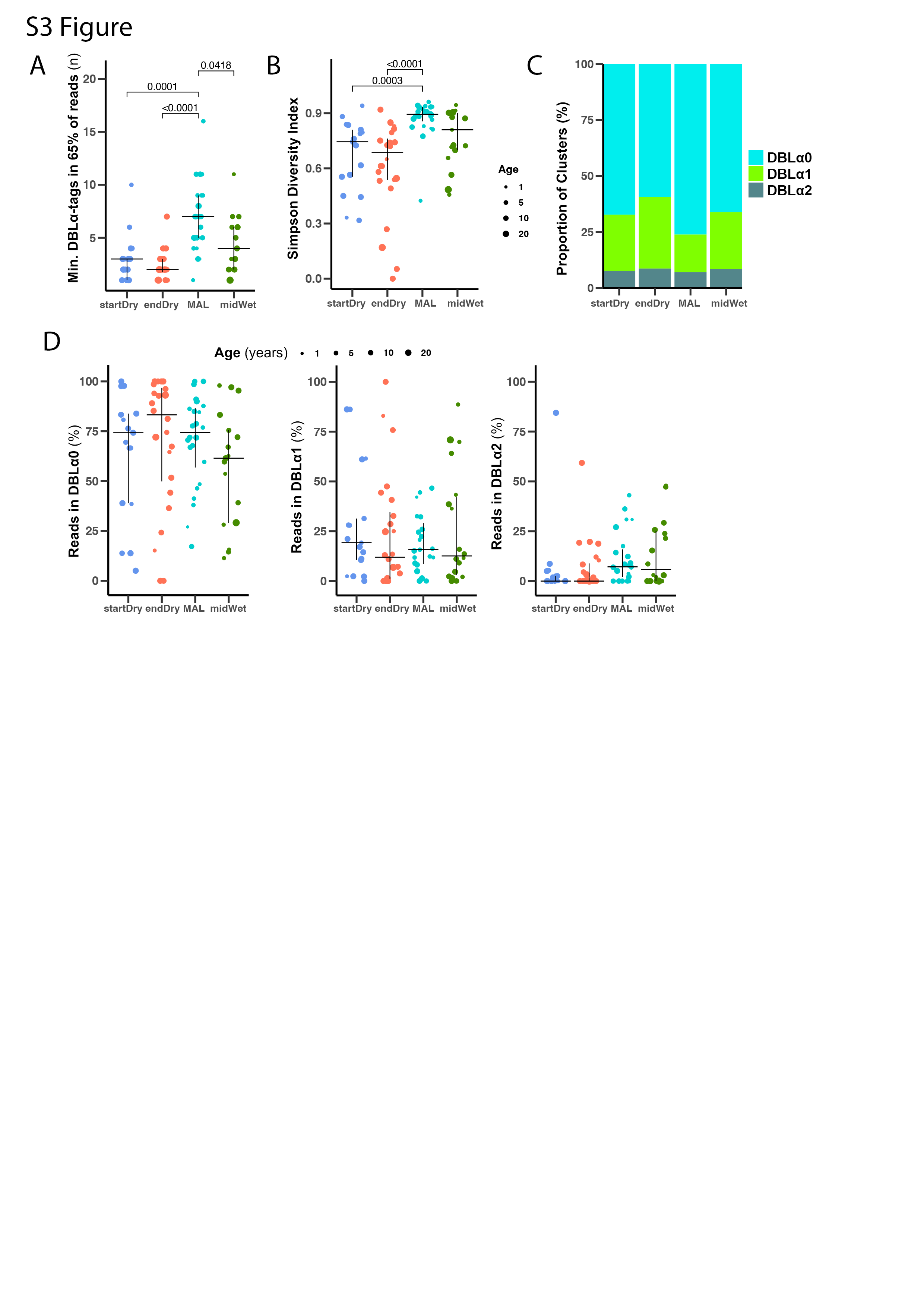

Supplement: S3 Fig — (A) Minimum number of DBLα-tags in 65% of reads in 82 samples from clinical malaria cases and asymptomatic infections (startDry = 17, endDry n = 24, MAL n = 24, midWet n = 17). Median and IQR are shown with dot size indicating participant age. Kruskall-Wallis test with Bonferroni multiple comparison correction (B) Simpson diversity index of read distribution in the same 82 samples. Median and IQR are shown with dot size indicating participant age. Kruskall-Wallis test with Bonferroni multiple comparison correction. (C) Proportion of DBLα-tags annotated to DBLα-subtypes. Annotation is based on the best Blast hit in an annotated var database (min e-value 1e-02). Shown is the proportion of clusters detected at each of the timepoints belonging to the three DBLα-subtypes. (D) Proportion of DBLα-tag sequencing reads mapping to DBLα-tag clusters annotated as DBLα0 (left panel), DBLα1 (middle panel) or DBLα2 (right panel) in 82 samples across the four timepoints (startDry = 17, endDry n = 24, MAL n = 24, midWet n = 17). Annotation as described above, median and IQR are indicated on the plot with dot size showing participant age. Kruskall-Wallis test with Bonferroni multiple comparison correction. (TIF) [file ppat.1013210.s003.tif]

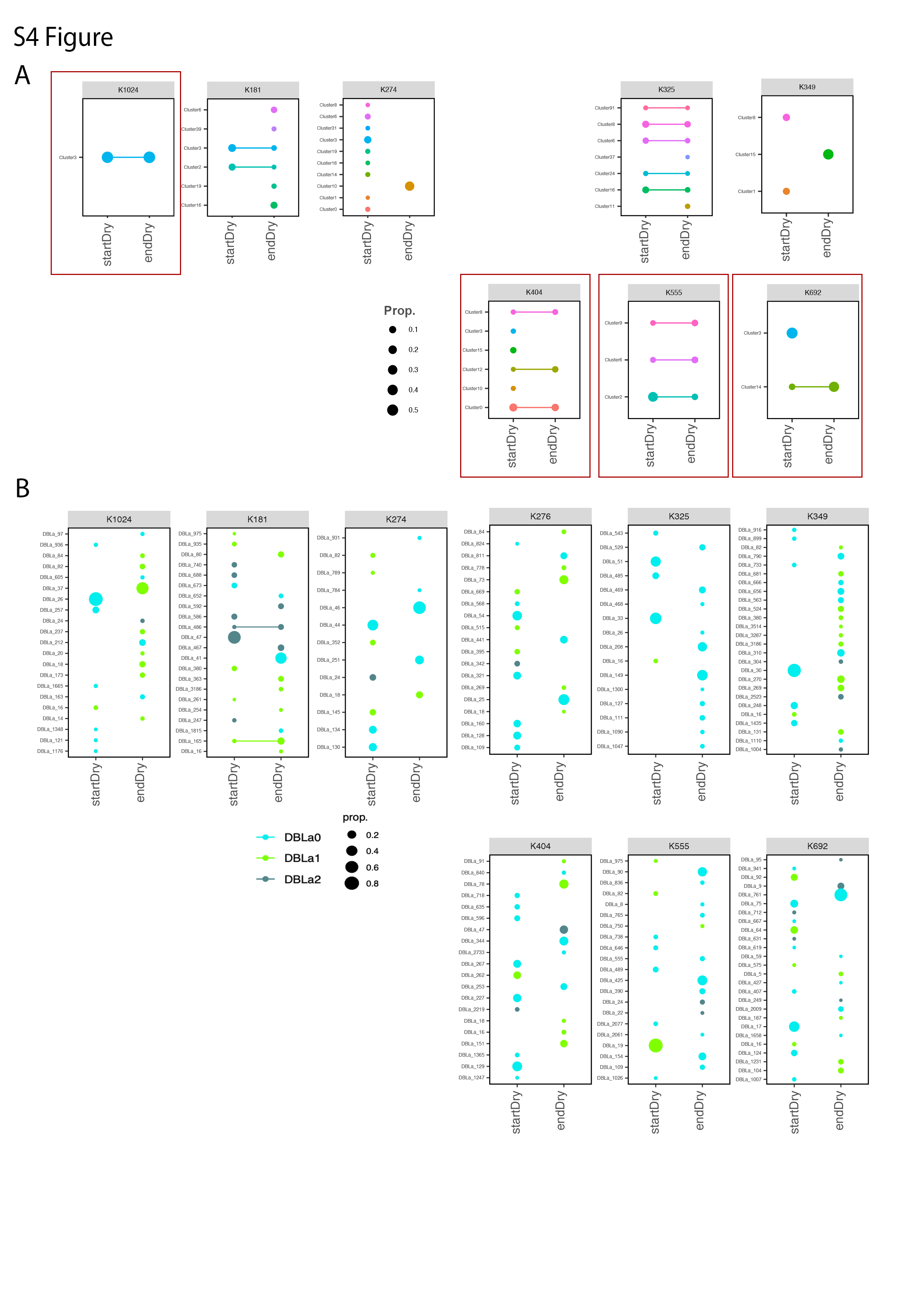

Supplement: S4 Fig — (A) AMA1 haplotype sharing between paired samples of 8 study participant at the beginning (startDry) and end of the dry season (endDry) 2019. Each box corresponds to a study participant, participant ID is shown in the gray shaded area. Dots represent a haplotype, identifiers are listed on the y-axis, with dot size indicating cluster abundance as proportion of reads. Clusters present at both timepoints are connected by a line. Two replicates per sample, haplotypes were clustered by 0.989 similarity and had to be present in both replicates. Red boxes indicate samples were all haplotypes present at the end of the dry season are also detected at the season’s start. (B) DBLα-tag sharing between paired samples of 9 study participants at start and end of the 2019 dry season, each box corresponds to a study participant with ID shown in the gray shaded area. Dots represent a DBLα-tag, shared tags between timepoints are connected by a line. Color indicates annotation by best BLAST hit (min value 1e-02) in an annotated var database, abundance as proportion of reads in the sample is shown as dot size. Samples of study participants are shown in the same position in A and B panels. (TIF) [file ppat.1013210.s004.tif]

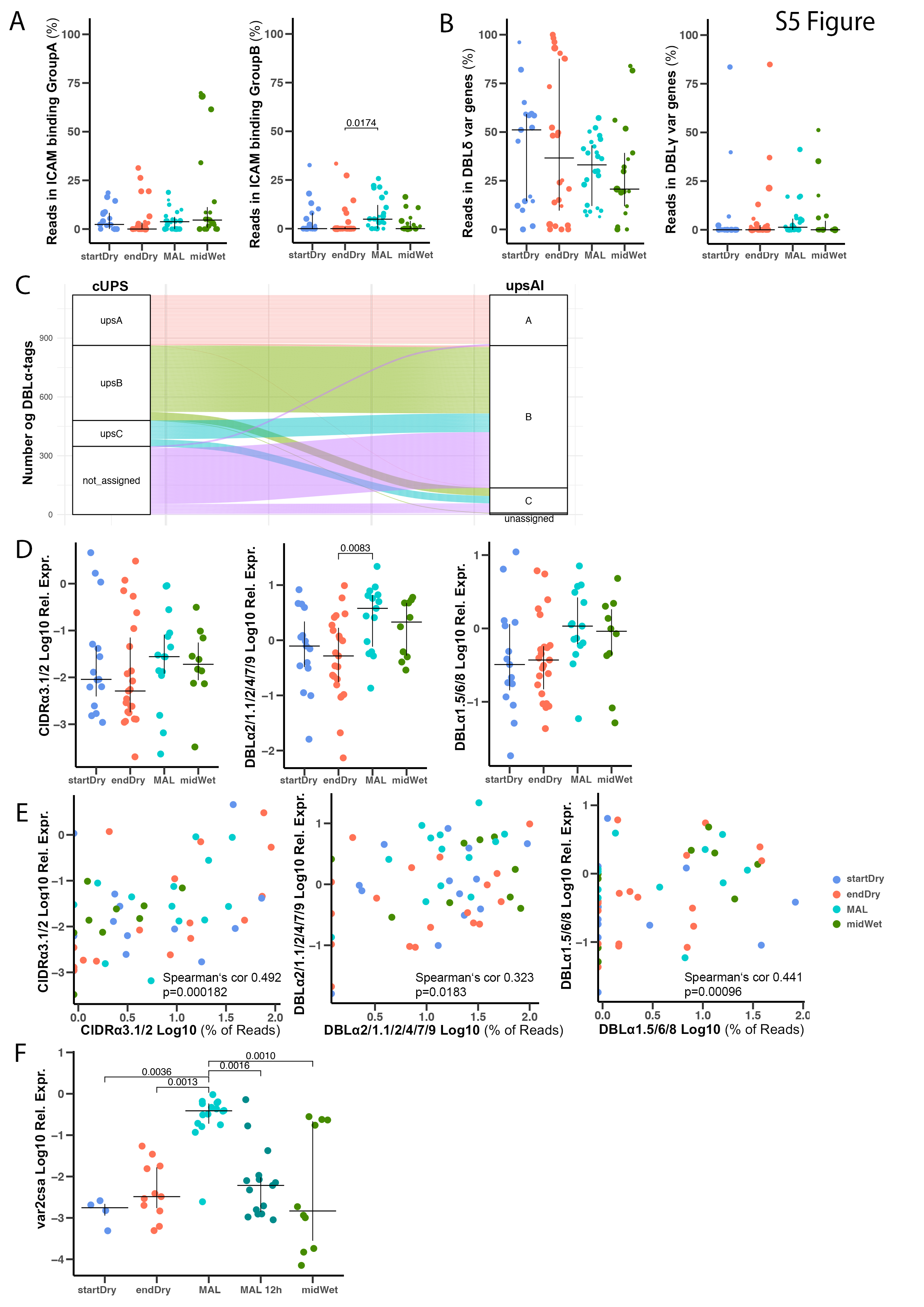

Supplement: S5 Fig — (A) Expression of putative ICAM1-binding var genes in 82 samples (startDry = 17, endDry n = 24, MAL n = 24, midWet n = 17). var genes with predicted CIDRα1 plus DBLβ1/3, were assigned to group A and CIDRα2 – 6 plus DBLβ5 domains to group B ICAM-1-binders. (B) Expression level of DBLδ and DBL𝛾 containing var genes in the same samples. (C) Sankey plot of comparing annotation of DBLα-tags with cUPS and upsAI. (D) Relative expression of different var-gene domain types quantified in 54 samples (startDry n = 15, endDry n = 24, MAL n = 15, midWet = 10) by qPCR normalized to a housekeeping gene (Fructose-bisphosphate aldolase, PF3D7_1444800). (E) Correlation of relative expression of different var-gene domain types measured by RT-qPCR in 54 samples (startDry n = 15, endDry n = 24, MAL n = 15, midWet = 10) and proportion of reads in DBLα-tag clusters belonging to var-genes with the equivalent domain types based on Varia prediction in the same samples. Color indicates timepoint. (F) var2csa expression in clinical and asymptomatic infections (startDry n = 4, endDry n = 11, MAL n = 15, Oct n = 10) and clinical malaria cases after short time of in vitro culture (MAL 12h, n = 15). B, C, E and G Kruskall-Wallis test with Bonferroni multiple comparison correction. (TIF) [file ppat.1013210.s005.tif]

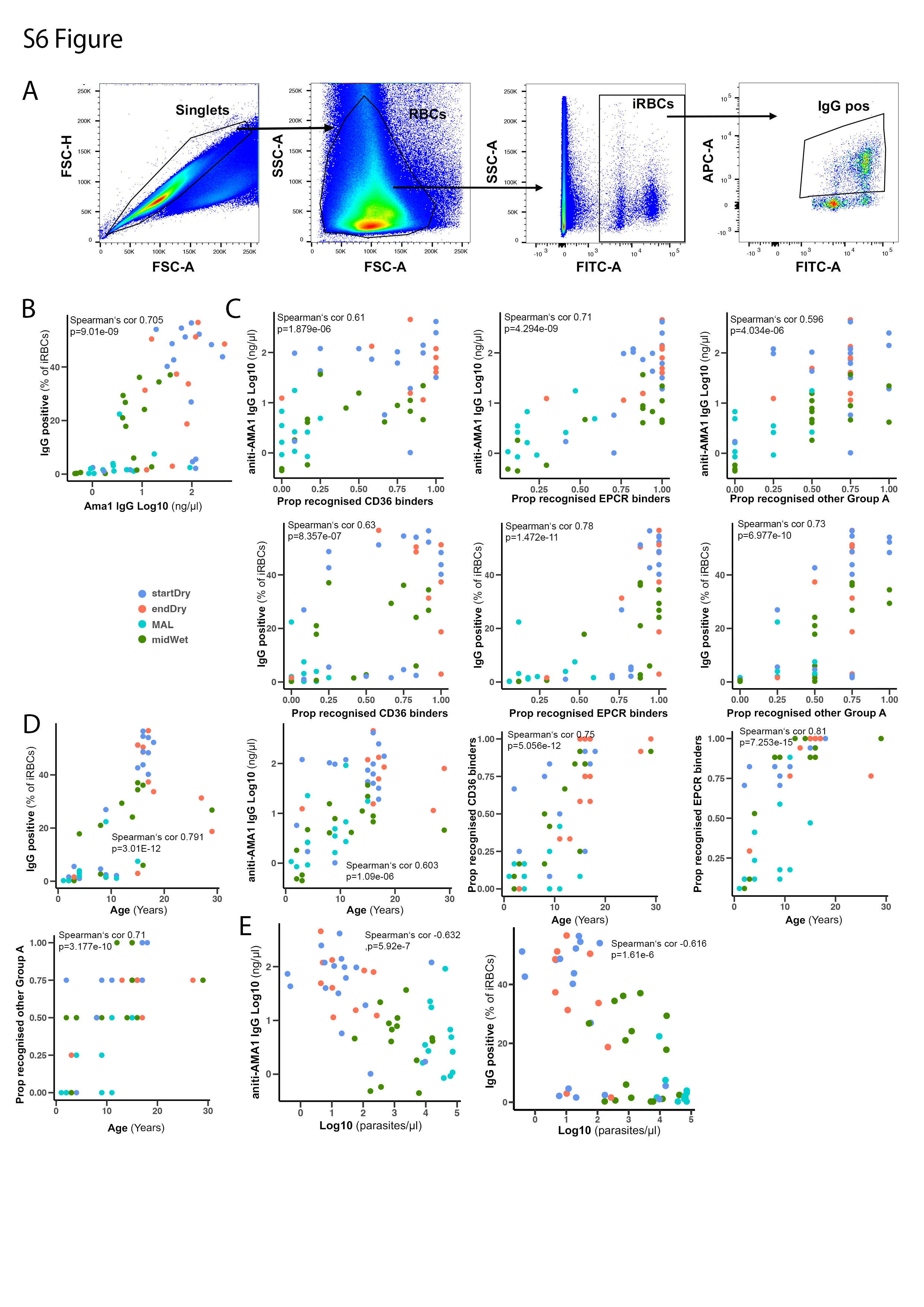

Supplement: S6 Fig — (A) Gating strategy of surface recognition assay (SRA) with FCR3 iRBC incubated with plasma of pooled plasma of Malian individuals and stained with SYBRgreen, first gated on single cells based on forward and side scatter (top two small plots each panel) and then on infected RBC based on SYBRgreen staining in the FITC channel (bottom small plot each panel). Surface recognition was identified by staining with an anti-human IgG APC antibody and detected in the APC channel (big plot each panel). (B) Correlation of humoral immunity measured by SRA (left) and AMA1 ELISA (right) in plasmas from dry and wet season (startDry 19 n = 17, endDry n = 10, midWet n = 17, MAL n = 10) to participant age with color indicating timepoint. (C) Correlation of humoral immunity measured by and AMA1 ELISA (top) and SRA (bottom)in the same plasmas and breadth of recognition of var domains measured in Luminex. (D) Correlation between the different measures of humoral immunity and participant age. (E) Correlation of humoral immunity measured by AMA1 ELISA (left) and SRA (right) and parasitaemia. (TIF) [file ppat.1013210.s006.tif]

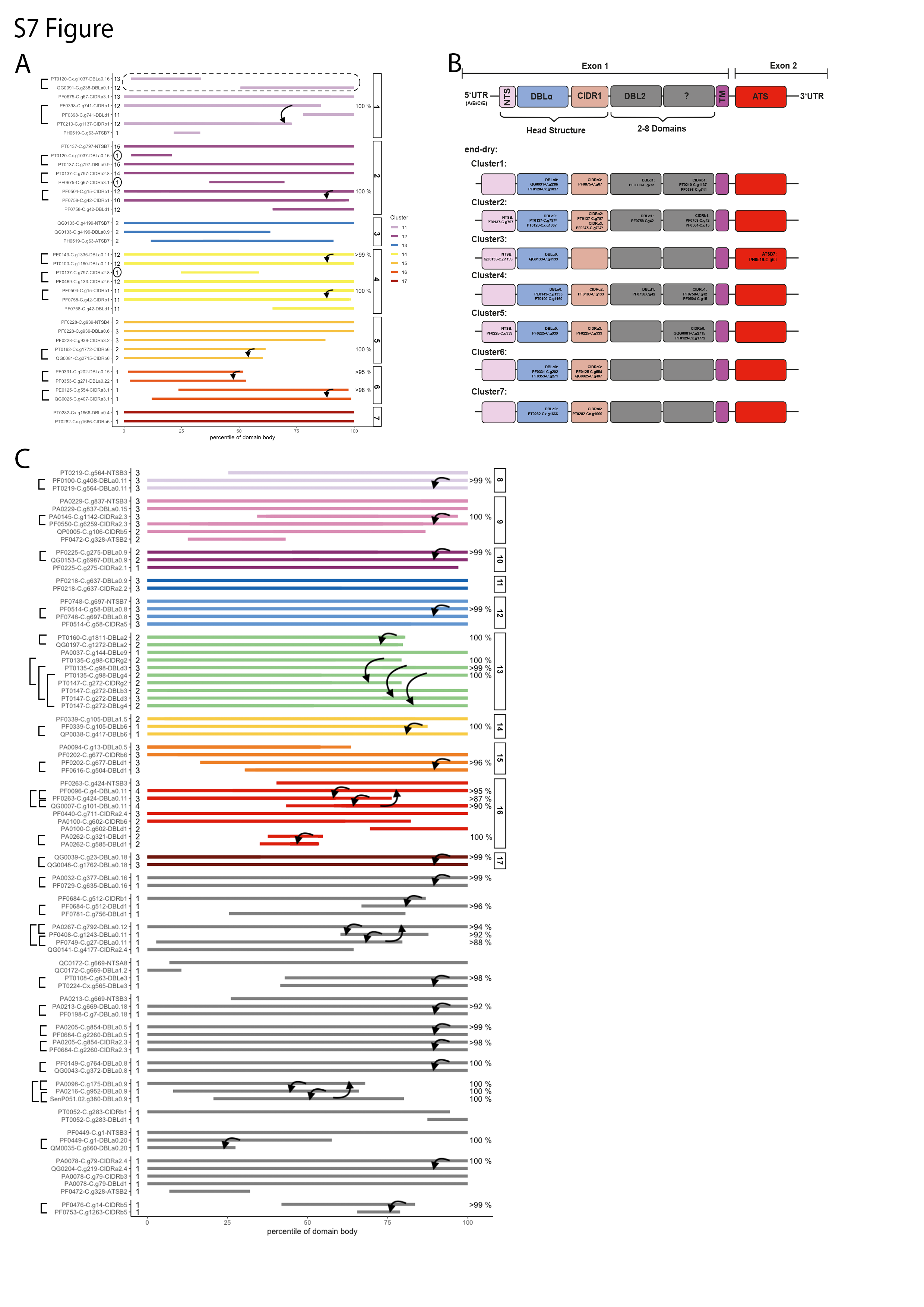

Supplement: S7 Fig — (A) Comparison of regions with sequencing coverage by smart-seq of an end-Dry season asymptomatic infection. Cells were clustered by var expression into 7 clusters indicated by color, and coverage of individual var domains in a reference database shown by cluster. The y-axis delineates identifiers of the individual domains with mapping reads, the x-axis shows sequencing coverage by region of the respective domains. Number of cells with coverage of a specific domain type is shown next to the sequence identifier. 11 instances of reads mapping to different reference domains of the same domain type in the same cluster were detected, highlighted by brackets on the plot. Overlapping regions with coverage in the same cluster (indicated by arrows) were compared by BLAST, sequence similarity of sequences in percent is shown on the right of the respective sequence. In cluster 1, we observed mapping to different regions of different sequences of the same domain type (indicated by dashed line). In cluster 2 and 4, mapping across the majority of cells was congruent, only a single cell showed a divergent expression pattern. (B) Proposed var gene domain architecture of clusters obtained by single-cell RNAseq of end-dry season sample. Top panel shows domain composition of var genes overall, below reference domains detected by single-cell RNAseq are listed in the corresponding positions for each cluster. (C) Equivalent plot to A describing var read mapping in a clinical malaria case. (TIF) [file ppat.1013210.s007.tif]
