# Supplementary material for: Plasmodium falciparum expresses fewer var genes at lower levels during asymptomatic dry season infections than clinical malaria cases
Source: PLoS Pathog. 2025 Jun 10;21(6):e1013210. doi: 10.1371/journal.ppat.1013210 (PMC12151486; doi:10.1371/journal.ppat.1013210)
Supplement: S4 Table — (DOCX) [file ppat.1013210.s011.docx]

**Key Resources Table**

| **Resource** | **Designation** | **Source** | **GeneID** | **Additional information** |
| --- | --- | --- | --- | --- |
| Cell line | *P. falciparum* FCR3 | PMID: 781840 |  |  |
| Primer | varATS For | PMID: 25734259 |  | CCCATACACAACCAAYTGGA |
| Primer | varATS Rev |  |  | TTCGCACATATCTCTATGTCTATCT |
| Probe | varATS Probe |  |  | 6-FAM-TRTTCCATAAATGGT-NFQ-MGB |
| Primer | AMA-OF |  |  | GCTGAAGTAGCTGGAACTCAA |
| Primer | AMA1-R |  |  | TTTCCTGCATGTCTTGAACA |
| Primer | Ama1F+a |  |  | ACACTCTTTCCCTACACGACGCTCTTCCGATCT-CCATCAGGGAAATGTCCAGT |
| Primer | AMA1R+a |  |  | GTGACTGGAGTTCAGACGTGTGCTCTTCCGATCT-TTTCCTGCATGTCTTGAACA |
| Primer | P61 For |  |  | TGTACCACCAGCCTTACCAG |
| Primer | P61 Rev |  |  | TTCCTTGCCATGTGTTCAAT |
| Primer | P90 For |  |  | TCAATTTGATAAAGTGGAACAATTC |
| Primer | P90 Rev |  |  | GCGTTGTTTAAAGCTCCTGA |
| Primer | varF_dg2 |  |  | TCGTCGGCAGCGTCAGATGTGTATAAGAGACAG-GCAMGMAGTTTYGCNGATATWGG |
| Primer | Brlong |  |  | GTCTCGTGGGCTCGGAGATGTGTATAAGAGACAG-TCTTCDSYCCATTCVTCRAACCA |
| Primer | Ruf6A Fwd |  |  | AAGCTGCCTCAGTAGCCCA |
| Primer | Ruf6A Rev |  |  | AAAAATTGCGCCACCCCC |
| Primer | Ruf6B Fwd |  |  | TGTACCACCAGCCTTACCAG |
| Primer | Ruf6B Rev |  |  | AAAAATTGCGCCGCCCCC |
| Primer | DBLα1.5/6/8_fwd 1 |  |  | TGGTWYRANGAATGGGCAGAAGA |
| Primer | DBLα1.5/6/8_fwd 2 |  |  | TGGTTYGAGGAATGGAGTGAAGA |
| Primer | DBLα1.5/6/8_rev 1 |  |  | TTTTAGTACAATCATAACCATCACCA |
| Primer | DBLα1.5/6/8_rev 2 |  |  | GATTTGTTTTWTTACAATCGTAACCCTC |
| Primer | DBLα1.5/6/8_rev 3 |  |  | ACAATCCTCACCATCACCACTACAAT |
| Primer | DBLα1.5/6/8_rev 4 |  |  | CGTGATATATCTGTTTKAGTACAATC |
| Primer | DBLα1.5/6/8_rev 5 |  |  | GATCTGTTCGTTTACAATCGTAACCCTC |
| Primer | DBLα2/1.1/2/4/7/9_ fwd 1 |  |  | TGGTWYRANGAATGGGCAGAAGA |
| Primer | DBLα2/1.1/2/4/7/9_ fwd 2 |  |  | TGGTTYGAGGAATGGAGTGAAGA |
| Primer | DBLα2/1.1/2/4/7/9_ rev 1 |  |  | TACAATCATATCCATTAWGACTACAA |
| Primer | DBLα2/1.1/2/4/7/9_ rev 2 |  |  | TCACAATCGCATCCATTATGACTACAA |
| Primer | CIDRα3.1/2_fwd 1 |  |  | AHWWVCAAAAGACRTWCHATRATTT |
| Primer | CIDRα3.1/2_fwd 2 |  |  | AHWWVCAAAAGACRTTCAATCCT |
| Primer | CIDRα3.1/2_fwd 3 |  |  | ARAAAGTAAAGGATTATGTWGRTTT |
| Primer | CIDRα3.1/2_rev 1 |  |  | TTTTTGTTCTCCAATRTATRGAATC |
| Primer | var2csa_fwd |  |  | AATGGGACAAACAAAAAACAAAATATG |
| Primer | var2csa_rev |  |  | GCTGATATACATTCAGGATAATTTTC |
