# Supplementary material for: Plasmodium falciparum expresses fewer var genes at lower levels during asymptomatic dry season infections than clinical malaria cases
Source: PLoS Pathog. 2025 Jun 10;21(6):e1013210. doi: 10.1371/journal.ppat.1013210 (PMC12151486; doi:10.1371/journal.ppat.1013210)
Supplement: Attachment — Submitted filename: reSub c&K Response to Reviewers.pdf [file ppat.1013210.s013.pdf]

Point by point reply to the Reviewers' Comments:

Part I - Summary
Please use this section to discuss strengths/weaknesses of study, novelty/significance, general execution and scholarship.

Reviewer #1: In this manuscript, the authors investigated the expression of parasite virulence genes in a Malian cohort with seasonal malaria. In particular, they sought to explain the differences in circulating parasite stages between wet and dry season parasites observed in a previous publication, suggesting differences in cytoadhesion of infected cells as the cause. In this work, they included samples of clinical cases from the wet season and samples from asymptomatic patients at the beginning and end of the dry season, but also from the middle of the rainy season as an additional control. First, the cohort is characterized in terms of parasite prevalence, parasitemia, complexity of infection, age and later the degree of immunity and these parameters are linked to the expression pattern of the var genes of the parasites, which they determined using an expressed sequence tag approach (DBLa-tag) in conjunction with previously published bioinformatics tools to predict the full-length encoded PfEMP1 protein (Varia) and its var group identity (cUPS) by its upstream region. In addition, they performed SMART scRNAseq on one isolate each from the wet and dry seasons, a method that generates reads from the full-length var genes and enables the determination of the composition of the encoded domains. In principle, methods and tools used are well established in the field, but their application to a seasonal malaria cohort is novel. From their data, they conclude that the diversity of var gene expression is limited in asymptomatic cases, with the samples at the end of the dry period showing the most extreme phenotype of very homogeneous expression of mostly a single variant and the clinical cases representing the other extreme with very diverse var gene expression. In addition, the overall level of var gene expression is reduced in asymptomatic cases. But apart from finding more EPCR-binding variants associated with severity in clinical cases, no difference in predicted binding phenotype or var group could be detected that could account for the reduced cytoadhesion.

Overall, this is a very convincing study that confirms an earlier publication by Warimwe et al. 2013 (PMID: 23922996), in which the authors described the same trend of asymptomatic infections having parasites with less diverse var expression, lower parasitemia, being older and having a higher proportion of infected erythrocyte surface-recognizing antibodies compared to non-severe and severe cases. However, as this older data set was generated by DBLa tag PCR with cloning and Sanger sequencing of a limited number of clones, the data presented is of significantly higher quality, and samples from the end of the dry season may even have a stronger “asymptomatic” phenotype. Especially, the controls included by the authors in the experimental design are very useful, such as the confirmation of DBLa-tag results with a subset of samples and genes using variant-specific qPCR primers, doing technical replicates or the exclusion of samples falling below a certain threshold for parasite RNA content to ensure comparability. The scRNA data strongly support the differences in var expression diversity between asymptomatic and clinical cases, although the overall level of var gene expression was not analyzed using this method, only a limited number of cells were analyzed and the data are likely to be difficult to grasp for non-var experts.

Reviewer #2: The submission from Ceesay and colleagues describes a detailed examination of var gene expression in individuals infected by P. falciparum in Mali. The authors analyzed samples at different points in the transmission season and in individuals that had different levels of anti-malaria immunity. The study site is particularly interesting due to the stark differences in transmission rates across the length of the year, with 6 months of intense transmission and 6 months of very little transmission. This provided the authors with the ability to investigate how var gene expression changes with the acquisition of immunity. They are also able to examine infections that they could be certain had not been recently obtained. In addition, they could compare symptomatic and asymptomatic infections. The authors obtained data indicating that as individuals acquire greater anti-PfEMP1 immunity, var expression levels fall, the number of var genes that are detectably expressed decreases, and symptoms similarly decrease. Many of these conclusions were anticipated and make sense base on current models of immunity, nonetheless validation by this study is valuable. The more provocative conclusions are that parasites might respond to anti-PfEMP1 immunity by reducing total var gene expression, something that has been hinted at in laboratory studies but not be described in the field. In addition, the authors found no correlation with PfEMP1 type and dry vs wet season transmission, which contrasts with some previous predictions. Overall, this is a valuable dataset from

an interesting study that will be valuable to researchers interested in the dynamics of PfEMP1/var expression during natural malaria transmission.

Reviewer #3: The study "Plasmodium falciparum expresses fewer var genes at lower levels during asymptomatic dry season infections than clinical malaria cases" by Ceesay et al. investigated var gene expression in asymptomatic infections and clinical malaria cases among Malian children during dry and wet seasons. Sequencing and bioinformatics analyses revealed that parasites from asymptomatic infections expressed fewer var genes, with transcripts dominated by one or a few var types, while clinical cases exhibited broader var gene expression at lower levels. Parasites from asymptomatic carriers expressed a mix of CD36- and EPCR-binding PfEMP1, with CD36-binding variants dominating in non-severe cases. The findings suggested that host immunity was key in limiting var transcript diversity.
The manuscript is well written, uses state-of-the-art techniques and provides novel insights into malaria pathogenesis and persistence. It thus is of interest for a broader community. I only have some minor remarks.

Part II – Major Issues: Key Experiments Required for Acceptance
Reviewer #1: 1. My main concern is the claim that the overall level of var expression decreases during the dry season to sustain long-term infections. This data point is based solely on qPCR results using an ATS primer designed for diagnostic purposes, which according to the original paper (PMID: 25734259) only covers 40% of all variants and mainly B-type var genes (17 out of 19 genes in 3D7 that can be amplified from 3D7 according to Primer3 Blast). Instead of a decrease in the total amount, this could also be interpreted as a shift towards other var groups that are not covered by the primer. Especially since clinical cases are younger and less immune, I would assume that they express many B-types and may therefore be better covered by the primer. Therefore, I would be cautious to draw conclusions about the total var value solely based on this primer. Do the authors have additional data to support this point? I suggest backing this up with another approach such as the scRNAseq data. Can the authors infer anything from their scRNAseq data, e.g. more var reads in the malaria case, higher coverage of the LARSFADIG motif (as in PMID: 38270586)? Otherwise, this needs to be toned down.

In the course of this work, we have tested the varATS primers from Hofmann et al. (2015), which we include in Fig. 4 of the manuscript; and those reported by Guillochon et al. (2022) that would in principle afford better coverage of the var *family. However, we could never make the latter work. We obtained a detailed protocol directly from the authors through personal communication, but detected qPCR amplification product only when >100ng of genomic DNA were used as template, making the primers unsuitable for analysis of low-parasitaemia asymptomatic infections.*
In support of decreased overall var *expression in asymptomatic infections we have also quantified* expression of *ruf6_ a* ncRNA expressed through RNA Pol III that promotes *var* gene expression.
The bulk RNAseq approaches done in Andrade et al. 2020 paper and suggested by the reviewer to apply to the current scRNAseq data are in our view of limited power due to the very small number of cells available and low read depth, as we now mention it in the discussion lines 484-486.
All together, we agree with the reviewer that we cannot completely disentangle whether lower overall var *expression takes place, or if switch to* var *types that are less covered by the primers used promotes the differences detected. In agreement, we now added a sentence to lines 478-480 of the revised manuscript: "…Nevertheless, the varATS primers coverage across the var family is only ~40%, leaving the possibility of a skewed, and not reduced* var *expression" although we did not observe a skew away form B types in DBLα-tag sequencing data.*

2. The prediction of the upstream region and associated var gene groups from DBLa tags using cUPS is not reliable, especially the distinction between B and C types (accuracy of 48.3 %) (Preprint Tan et al 2023; doi: 10.1101/2023.11.05.565723). Thus, it remains unclear whether a specific var group signature is associated with asymptomatic infections, as this question cannot be answered with the chosen approach and requires the development of new methods. Unfortunately, this technical limitation is not discussed.

We agree with the reviewer that the cUPS tool used to track UPSC var genes is imperfect. To complement this analysis, we have now included results obtained with the new tool upsAI (Thomas Otto personal communication and https://github.com/sii-scRNA-Seq/upsAI), which improved the

overall var type calling accuracy using the same training datasets (74% accuracy with cUPS and 84% with upsAI), and even higher with an improved training dataset. Although differentiation of ups B and C remains challenging, the overall improved accuracy of upsAI increases confidence of UPSC var calling.
With the upsAI tool fewer DBLα-tags of our samples remain unassigned. Most upsA vars *maintained their type, while upsB and C types disagreed more, particularly on what the cUPS tool called upsC. The numbers of var types upsA, B and C identified in our dataset with the two tools are:*

cUPS:
A: 22.9% (n = 257)
B: 34.2% (n = 383)
C: 11.7% (n = 132)
unassigned: 31 (n = 348)

upsAI:
A: 22.9% (n = 258)
B: 64.1% (n = 726)
C: 12.1% (n = 128)
unassigned: <1% (n = 8)

Using the var *types identified with* upsAI *our data continues to show no statistically significant difference of* var *types expressed between the timepoints analysed.*
We adjusted the result section accordingly in lines 270-275 of the revised manuscript, and edited Fig. 3 and S Fig, 5 to include the new data.

Part III – Minor Issues: Editorial and Data Presentation Modifications
Please use this section for editorial suggestions as well as relatively minor modifications of existing data that would enhance clarity.
Reviewer #1: Unfortunately, the lines in the manuscript are not numbered, which makes it somewhat difficult to refer to specific text passages.
Apologies, these have now been added.
1. Abstract: There is evidence for older stages in the circulation, which might indicate reduced cytoadhesion – as stated at the end of intro –, not vice versa as stated in the abstract: "(…) shows reduced cytoadhesion of infected erythrocytes, evidenced by the circulation of further developed parasite stages (…)".
The reviewer is correct and we have reversed the arguments accordingly.
2. Abstract: "However, by RNAseq and qRT-PCR we also observed significantly higher total var transcript levels in malaria cases compared to asymptomatic carriers." As far as I have seen, var levels were only assessed by RT-qPCR, not by RNAseq. Please clarify!
The reviewer is correct, we removed the mention to RNAseq from the abstract sentence.
3. Please add reference PMID: 14651636 for var subgrouping (together with Lavstsen 2003, ref29) in the introduction.
We have added the suggested reference.
4. Please add studies on var gene expression in asymptomatic infections in the introduction and discussion: PMID: 15122533 (even a longitudinal study) and PMID: 23922996. Please specify which studies suggest a link between asymptomatic infections and varC expression.
We have reworded the introduction (lines 86-92 of the revised manuscript) to more clearly describe prior studies, including those suggested by the reviewer.
5. To further improve the quality of the assumptions that can be made, the duration of infection should be assessed as an additional variable. Can the authors use regularly collected dried blood spots/venous blood samples and determine the COI/genotype and assess how long the individuals were infected and also correlate this with var gene expression, parasitemia, etc. as they have done in PMID: 39284949?
Although we agree with the reviewer that it would be interesting to evaluate the relation of var *gene expression and infection duration, we believe that the size of the analysed groups is not large enough to allow meaningful associations, especially in the wet season asymptomatic infections group that should include shorter and longer infections.*
In S Fig. 4 we present the paired startDry and endDry season samples available in our analyses for which we can be confident that infections persisted the 5 to 6 months of the dry season given the absence of mosquitoes and transmission during that time. For the clinical malaria cases, we and others have reported that these are mostly the product of recent infections and hence of short duration (Babiker 1998, Mueller 2012, Hofmann 2015, Andrade & Carrasquilla 2024). For the

asymptomatic infections of the wet season (midWet), although monthly filter paper and treatment data were collected for the majority of individuals, we did not evaluate COI or duration of infection of the longitudinal samples in the course of the work presented in this manuscript, but can from the clinical records determine that 6 of the 17 midWet samples were treated in the preceding months and likely carrying shorter infection than the non-treated individuals, but amplicon sequencing would be required to establish infection length.

6. Parasites from clinical cases that had been cultured for 12 (DBLa-tag) or 16 hours (scRNAseq) served as controls in some experiments. However, the difference in the mean age of wet and dry season parasites is only about 6 hours, as shown in a previous publication (PMID: 35927582). Please comment!

In our 2020 publication of we calculated 6-hour difference with the maximum likelihood estimation (MLE) method by Lemieux et al. 2009 using bulk RNAseq data, but also report the presence of fully segmented schizonts and new ring-stages 12-24 hours earlier in dry season samples than in the clinical malaria cases in culture, so we would not be so strict as to say that only one of those analyses is correct. To make it clearer that the 12-16h in vitro culture shown in the current manuscript aimed to investigate the effect of cell cycle progression on var expression, and not only stage-match samples, we have edited the revised text in lines 176-177 accordingly.

7. It has already been shown that changes in var gene expression can occur during short-term in vitro cultivation in samples, although these cannot be predicted (PMID: 38270586). This could serve as an additional argument on page 6 (section before Figure 1).

Although the reviewer suggestion is valid, we believe that samples used in the article described have all gone through at least one full generation and reinvasion in vitro, rather than a few hours as in our case, so we would rather maintain our current version text.

8. Figure 1F: A Blant-Altman plot should be used to compare two methods, please replace.

We made the Blant-Altman plot suggested by the reviewer to compare the DBLα-tag sequencing and the qPCR, which is shown below, with all but two points falling within the ±1.96 standard deviation of the mean difference between the two methods. However, as the relative expression values obtained through DBLα-tag sequencing and qPCR do not have the same unit_ we are comparing relative expression to all var *reads in the DBLα-tag sequencing, and relative expression to a housekeeping gene in the qPCR, we do not feel that such presentation is the best to convey the data.*

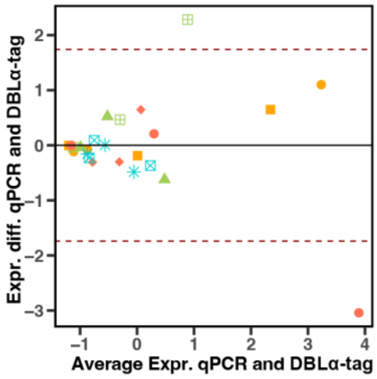

We believe that the current Fig. 1F better informs the reader that qPCR confirmed that DBLα sequences obtained with the tag sequencing were indeed present in those samples with the described proportional expression.

9. It would be helpful if the pseudotimes for the parasites analyzed by RNAseq could be provided to assess the homogeneity of the developmental age of the parasites within the asymptomatic and clinical sample.

We have added this information to Fig. 6A and the methods section. We did the staging using a P. falciparum *single cell atlas that produced in our laboratory and recently preprinted, which is now cited accordingly in line 357.*

10. Please provide details of the primers used in the study. Currently there is not even a reference in the methods section for the P61, P90 and no details at all for the qPCR primers used to validate the DBLa tag results.

We have added the references for P61 and P90 in method ***RNA extraction and cDNA synthesis*** *Section, and added a* ***qPCR validation primer sequences design*** *section to the methods also.*

11. On page 4 it is stated that RNA was assessed with two housekeeping genes to select samples

with sufficient parasite RNA. However, the "Methods" section (page 22) states that only P90/seryl-tRNA was used to select samples
We corrected accordingly and explained in the methods section that qRT-PCR was done with both primers, but selection was based on P90 CT values, as this was the reaction affording lower expression levels.

12. Please provide the formula for calculating the Simpson Diversity Index in the Methods section.
This was in the Legend of S Fig. 3 and has now been moved to the method section on line 618.

13. Figure 2: I would suggest showing Figure S3B instead of Figure 2C in the main figures, as the diversity index is based on both measurements, the number of clusters found and the proportions.
That was also a subject of debate among the authors when we assembled the original figures, but after discussing we opted to include the simplest information of Fig. 2C in the main figure as we believe this may more easily reach all readers.

14. Figure 3: Since expression of the non EPCR-binding A-type ("unknown A" binding phenotype) was assessed in qPCR (DBL1.5/6/8), I would suggest including the predicted CIDRb/g/d domains in panel A as well, especially since the binding phenotype has also been associated with severe disease.
Following the reviewers' suggestions we have added further detail on var genes with an unknown biding phenotype. We have modified S Fig 5A to allow the reader to distinguish between var1 *and CIDRb/g/d var genes which were previously grouped together in the unknown group. The plot is now shown in Fig. 3A. We do not see a difference in these genes between timepoints, neither as proportion of DBLα-tags (now Fig 3A) nor when considering % of reads shown in the plots below.*

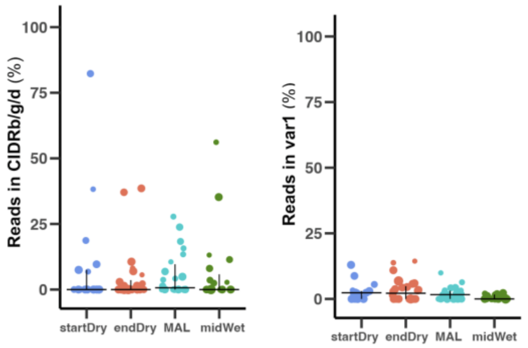

15. The overviews hidden in the supplement (Figure S3C, S5A & D) are easy to understand and should, in my opinion, be included in the main figures – especially as there are no obvious differences between the samples.
S Fig. 3C is a sum up of main Fig. 2D, and we believe it is best to maintain the individual data shown in main Fig. 2D and keep the overview in the supplementary material, as we feel it would be redundant to have both in the main figure.
S Fig. 5A and revised S Fig. 5D we have moved to main Fig. 3 as suggested by the reviewer.

16. Recently, ruf6 expression was shown to be transcribed from PolIII and regulated by Maf1, and an increase in ruf6 and var expression was associated with increased plasma magnesium levels in symptomatic wet season samples (PMID: 38921824). Do you have any data from your cohort to support this study?
We agree with the reviewer that this is an interesting question, however, we do not have magnesium values from the samples used in our study.

17. Page 12: Ref29 is not the correct reference for the Luminex assay
We have fixed it.

18. To improve the flow of the manuscript, I would suggest moving the section on the assessment of immunity to the first chapter on the description of the cohort, as it fits with the age and parasitemia curves and then the scRNA results are not so detached.
We also debated the best way to present our data and provide a good flow to our manuscript. Although we agree with the reviewer that the assessment of immunity could come connected to age and parasitaemia in figure 1, we think it would make it still patchy to allow it relation to var types later I the manuscript, so we would prefer to maintain the story line as it is.

19. Also, is there an assessment of immunity in this cohort of those who were able to overcome the infection compared to those who remained positive throughout the dry season or were negative at the beginning of the dry season?
Does the reviewer refer to comparing individuals with infections at the start of the dry season that clear them before the end of the dry season timepoint? this is an interesting question, that we have

however, not addressed.
20. The entire section on scRNA may be difficult to understand for non-PfEMP1 experts. The mapping of the sc reads against varDB, in which the genomic sequences of the var genes of the isolates studied are not deposited, was performed because no reference genomes are available. Why did the authors not generate a reference, either by sequencing gDNA or by de novo assembly of var transcripts from RNAseq reads? At the very least, I would recommend providing schematics for each cluster with the putative domain composition, which would also show that different regions of a domain correspond to different domains in the database.
We have added a scheme to revised S Fig. 7 with proposed domain compositions for cluster 1, 2 etc.; and we added a sentence in lines 360-361 to clarify that de novo *assembly was not done due to limited sequencing coverage.*
21. Figure 6B&C: What is the difference between dots that are filled and others that contain a plus? I would suggest drawing vertical lines between the clusters.
The reviewer is correct that information on crossed and filled dots was missing, these refer to ex vivo and cultured cells, and that information has now been added that to the revised figure legend.
22. Please comment if you have seen var-null or multiple var-expressing cells as described in the preprint https://doi.org/10.1101/2024.03.08.584127.
We agree with the reviewer that this is an intriguing question, but believe that the scRNAseq method used does not have the necessary depth to make confident conclusions, and we now make that clear in the manuscript discussion.
Have you found any other expression differences apart from the var genes?
We have so far not investigated gene expression other than the staging of parasites and the var family, and although interesting think this is out of the scope of the current manuscript.
23. The authors provide the scRNAseq data only on request, as indicated in the legends of Tables S2 and 3. The data should be included in the manuscript supplement or deposited in publicly accessible databases.
This was wrong in the tables' legends and has been removed, all data is on Edmond repository as described in the data availability section.
24. We already know from CHMI infections and another cohort study using the DBLa tag approach that naïve (and also more severe cases) and less immune African individuals ("non-controllers") express a higher number of var genes, which is restricted in individuals with higher immunity ("controllers"), this might be worth discussing (PMID: 31295334; PMID: 27070311; PMID: 33908865; PMID: 34340541; doi: https://doi.org/10.1101/2023.12.27.23300577)
We revised the manuscript discussion in lines 460-461 and now make mention to the manuscripts suggested where var expression was compared between more and less immune individuals.

Reviewer #2: I have no major criticisms for the authors. The present a large set of data, and while alternative methods of analysis and presentation are possible, I will leave that to the authors' discretion.
Minor, suggestions:

1. In all the Figures, the authors occasionally use yellow or light green text (or symbols) that are virtually impossible to read on a white background. They should adjust all the colors to ensure easy legibility.
We edited the colours used throughout the figures to avoid yellow and light green text and symbols.
2. The legend to Figure 1 is missing a description of panel E. The current legend includes descriptions of panels F and G but labels them E and F.
This has been corrected.
3. In the first sentence on page 4 the authors use the abbreviation MAL but don't define it until later in the paragraph.
This has been corrected.
4. On page 7, the authors annotate their DBL-tag sequences to DBLα types 0, 1 and 2. Please provide the readers with a brief description of what these types are and why this is interesting.
This has been added.

Reviewer #3: • The reference numbering starts at [2]
This has been corrected.
• Page 4, first line: The abbreviation MAL is used for the first time, but not defined. Later in the manuscript, MAL is defined several times or sometimes the abbreviation is not used at all but the full

term. MAL should be defined one time, when the it is used for the first time, but then MAL should be used consequently throughout the text.
We have corrected the first definition of MAL, but rather keep the definitions repeated several times to help the reader throughout the manuscript.
• Page 4, center part: "The individuals' age correlated negatively to parasitaemia (Spearman's cor -0.52, p= 8.99e-7) (S Fig. 1A)" These values cannot be derived from the graph. There is a correlation coefficient of -0.39 in the graph.
This was a mistake and has been corrected.
• Page 13, center part: "iRBC surface recognition , and..." There is an extra space.
This has been corrected.
• Page 13, last line of the text: The authors talk about a negative correlation, so it should be -0.71 instead of 0.71. (It is also incorrect in the graph.)
This has been corrected.
• Page 14, center part: Reference to Fig. 6a - it should be 6A.
This has been corrected.
• Page 19, line 4: Fig.4 - a space is missing here.
This has been corrected.
• Materials and Methods section: µ is italicized in some places and not in others.
This has been corrected, and all µ *are now not italicized.*
• Page 20, end of the first paragraph: One study exclusion criterion is a temperature of greater than or equal to 37.5 degrees? Does this make sense when the authors further write the following: Clinical malaria cases were defined as Plasmodium infection with parasitaemia >2500 parasites/µl blood and an axillary temperature >37.5°C?
This was incomplete, and we now added a sentence to clarify that axillary temperature >37.5°C was used as exclusion criteria at enrolment of the study participants only.
• Page 20, last paragraph: P. falciparum is not italicized.
This has been corrected
• Page 25, second paragraph: "Recombinant Pf3D7 AMA1." Incomplete sentence?
This has been corrected, and that half a sentence removed.
• Page 26, center part: flash-frozen instead of "flash-freezed."
This has been corrected
• Figure 1: The legend lacks a description of E; the panel labeled as E should be F, and what is labeled as F should be G. In the last two sentences of the legend, the statistical tests assigned to the panels are also incorrect.
This has been corrected
• Figure 2: The legend is missing an L in the last sentence at "individual."
This has been corrected.
• Figure S4: The legend states, "Red boxes indicate samples where all haplotypes present at the end of the dry season are also detected at the season's start." This cannot be seen. Does this only apply to A? Then what do the boxes mean in B?
The reviewer is correct that this is confusing, hence we removed the red boxed from panel B. And instead mention that data of study participants is shown in the same position in A and B panels.
• Figure S6: I believe the two panels of E do not appear in the text and also lack a legend. Does the legend for C and D match? The legend does not fit the figure.
This has been corrected.
